# The association between mental health and cognitive ability: Evidence from the Understanding Society survey

**DOI:** 10.1371/journal.pone.0318910

**Published:** 2025-10-08

**Authors:** Syka Iqbal, Muhammad Waqas, Min Hooi Yong, Ronan McGarrigle, Eleftheria Vaportzis

**Affiliations:** 1 Department of Psychology, University of Bradford, Bradford, United Kingdom; 2 Department of Accounting, University of Bradford, Bradford, United Kingdom; Imperial College London, UNITED KINGDOM OF GREAT BRITAIN AND NORTHERN IRELAND

## Abstract

**Background:**

The relationship between poor mental health and cognitive impairments in older age is well-established. Social engagement also influences cognitive ability. However, current research has not simultaneously accounted for the interplay between mental health, social interaction, and socio-demographic factors. This study addressed this gap by using a large, nationally representative dataset to examine the associations between mental health, cognitive ability, social interaction, and key socio-demographic factors.

**Method:**

In total, 7,685 individuals aged 65 or older were drawn from the UK Household Longitudinal Study Understanding Society. Cognitive abilities were assessed using self-reports and performance on five tasks (immediate and delayed word recall, subtraction, number series, and numerical ability). Mental health scores were derived from the General Health Questionnaire (GHQ-12). We controlled for social interaction, gender, ethnicity, educational background, marital status, number of children, and geographic location.

**Results:**

We found positive relationships between mental health and all measures of cognitive ability except performance on subtraction, and number series tasks. These relationships remained after controlling for social interaction. Socio-demographic factors that contributed to the relationship between mental health and cognitive ability included being White and having higher education. Being male predicted better performance on numerical tasks, while being female, married or divorced predicted better performance on the verbal memory tasks.

**Conclusion:**

We examined a wide range of cognitive domains using a large, nationally representative dataset. Overall, our findings provide novel insight into the relationship between mental health and cognitive abilities. This relationship persists when controlling for social interaction alongside socio-demographic factors in older adults, underscoring the importance of addressing these factors in policies and interventions for healthy ageing. Interventions should promote access to education, cognitive stimulation, and inclusive mental health services tailored to older adults.

## 1. Introduction

Older adulthood is a time of multiple changes in the cognitive, functional and social domains [1–3]. In particular, older adults are shown to be at increased risk of cognitive decline, loneliness, poor health and socio-economic deprivation [4], and these factors are associated with poor mental health outcomes such as depression and dementia [5]. There is a need for a better understanding of the determinants of cognitive decline and poor mental health.

Providing appropriate care to older adults is a global challenge. With longevity increasing steadily, there is a pressing need to better understand ageing populations and the relationship between cognitive decline and mental health. Maintaining cognitive abilities into old age is crucial for coping with chronic disease symptoms, quality of life, performing self-care, adhering to medication instructions and promoting independence in older adults [6]. Although cognitive function is a concern for older adults, cognitive decline is not inevitable in healthy ageing with research suggesting that the different domains decline at different rates [7]. For example, cognitive abilities such as vocabulary skills are resilient to the ageing process and may even improve gradually over time [8].

The current study aimed to examine the associations between cognitive ability and mental health. We employed a series of cognitive tests in a large nationally representative sample of older people in the UK, using the Wave 3 United Kingdom Health Longitudinal Survey data (UKHLS) to provide insights into the specific cognitive functions associated with poor mental health in older age. This dataset allowed us to conduct a highly-powered statistical analysis and control for social interaction and socio-demographic factors such as gender, ethnicity, educational background, marital status, and number of children, to investigate the relationships between mental health, cognitive function, and social interaction in older adulthood. Rather than testing specific hypotheses, this exploratory study aimed to shed light on the complex and potentially bidirectional relationship between mental health and cognitive decline in older adulthood.

Although previous studies have examined the relationship between mental health and cognitive decline in older adults, existing studies have not simultaneously examined the interaction between mental health, social interaction, and socio-demographic factors. For example, Kang [9] explored age as a moderator between psychological distress and verbal fluency, while Kang [10] examined psychological distress as a mediator between neighbourhood social cohesion and cognitive performance. These studies underscore important pathways but do not account for the simultaneous influence of social interaction and a broad range of socio-demographic variables. This study addressed this gap by using a large, nationally representative dataset to examine the associations between mental health, cognitive ability, and social interaction, controlling for multiple key socio-demographic factors. This comprehensive approach can provide novel insights into how mental health and the interplay of these variables shape cognitive ageing. This paper identified which factors most strongly relate to cognitive resilience in later life.

## 2. Literature review

Bauermeister and Bunce [11] found that the relationship between mental health and cognitive decline varied within individuals, affecting certain cognitive functions more than others. For example, there were significant impairments in executive function and processing speed, while domains like vocabulary remained stable. This within-person variability suggests that cognitive decline in older adults is a selective process with specific functions potentially impacted more than others by mental health changes.

Numerous studies have established a correlation between poor mental health and cognitive impairments in older adulthood, supporting our focus on mental health as a key determinant of cognitive ageing. Gallagher et al. [12] conducted a study involving 7,610 older adults, of whom 1,133 (14.9%) reported clinically significant depressive symptoms at baseline and subsequently exhibited pronounced cognitive impairments. The study specifically examined older adults, so no direct comparisons were made with younger populations. Despite evidence of the relationship between mental health and cognitive decline in recent years, a gap remains in understanding specific cognitive functions that may be impacted by declining mental health. These gaps are further compounded by the ambiguity surrounding the relationship between depression and cognitive outcomes. For example, depression has been shown to negatively impact specific cognitive domains, such as episodic memory, executive function, working memory and processing speed [13–15]. However, not all research has been consistent with regard to the effects on isolated cognitive functions. For example, research showed that rates of depression were negatively correlated with incidental memory, executive function (cognitive processes such as planning and problem solving) and overall functionality (the ability to perform tasks and daily activities) [16] highlighting the nuanced relationship between mental health and cognition.

The relationship between mental health and cognitive decline may also be related to social network size and feelings of loneliness. The lack of significant connections and social integration is associated with barriers to visiting friends and fosters an increase in feelings of loneliness [17]. In a longitudinal study of 3,777 older individuals assessed at baseline, it was found that those who received more support throughout their lifetime, irrespective of the type of support they received, they had a 55% reduced risk of dementia and a 53% reduced risk of Alzheimer’s disease [8]. This indicates that strong social networks and a robust support system are critical for mental health in older adults. Furthermore, a qualitative study involving 59 participants found the central component of loneliness is a lack of connection with others [18]. Harada et al. [8] highlighted the importance of understanding normal cognitive ageing and the factors that can mitigate age-associated cognitive decline, such as cognitive reserve and lifestyle interventions. They found that while abilities such as processing speed and episodic memory tend to decline with older age, language and vocabulary remain relatively preserved. These preserved domains are more likely to be influenced by mental health status. Harada et al. also found that the relationship between loneliness and mental health was bi-directional; mental health conditions reduced the capacity to socialise with others and led to withdrawal from social activities. On the other hand, reduced socialisation led to a decline in mental health which precipitated further isolation, compounding the downward spiral. There are many possible factors that could influence the degree of loneliness in individuals, but having friends has been shown to minimise feelings of loneliness, and thus improve mental health.

Crucially, much of the research on loneliness and reduced cognitive ability has not accounted for the roles of mental health and socio-demographic factors. Seshadri et al. [19] estimated the ‘remaining lifetime risk’ of developing Alzheimer’s disease in individuals aged 65. They revealed a substantial gender disparity, with females exhibiting a twofold higher risk (12%) in comparison to males (6.3%). Additionally, Kuiper et al. [20] examined the associations between social network size, loneliness, and cognitive performance using neuropsychological tests measuring four cognitive domain scores of processing speed, interference control, verbal memory, and working memory in a sample of 378 depressed older adults. The study identified a moderate negative relationship between loneliness and working memory capacity. In another study, depressed older adults with lower education and income were found to have an increased risk of loneliness [21]. Reduced opportunities for social interaction may have contributed to this association. Other research has highlighted context-specific factors (e.g., number of children) which reflect changing demographics, including declining fertility rates. For example, Ajrouch et al. [22] highlighted the central role of children in providing informal support to older adults. Other important socio-demographic factors to consider include education, gender and number of children, as part of a complex system that more accurately captures the nuanced relationship between cognition and mental health [23].

Recent research has highlighted sex-based differences in cognitive trajectories in later life. Kheloui et al. [24] found differences in verbal memory and spatial processing between men and women across the lifespan, supporting previous evidence of gender-specific vulnerabilities or advantages in specific cognitive domains. These findings highlight the importance of including gender as a key socio-demographic variable in cognitive ageing research to enable a more comprehensive picture of factors influencing age-related changes in cognition.

Studies using data from the UK Household Longitudinal Study explored the complex interplay between psychological distress and cognitive performance in older adults. Kang [9] showed that age moderates the association between psychological distress and verbal fluency, highlighting differential vulnerability across age groups. Furthermore, Kang [10] found that psychological distress mediates the relationship between neighbourhood social cohesion and cognitive outcomes, highlighting the importance of contextual social factors. Building on these findings, our study extends the literature by simultaneously examining mental health, cognitive ability, and social interaction within a nationally representative UK sample. Unlike prior work that focused on either moderation or mediation in isolation, we integrate socio-demographic moderators (e.g., gender, education, marital status), assess multiple cognitive domains, and critically evaluate the bidirectional relationship between social engagement and psychological wellbeing. This multifactorial approach offers a more comprehensive understanding of cognitive ageing and its psychosocial determinants.

## 3. Materials and methods

We completed secondary analysis of de-identified cross-sectional data from Wave 3 of the UK Household Longitudinal Study (UKHLS; 25). We obtained the data from the Understanding Society website (www.understandingsociety.ac.uk). In this paper, we summarise the key aspects of the development of the survey and its methodology; a complete account can be obtained from several other reports [25–27].

### 2.1. Participants

UKHLS is a longitudinal study that interviews all members of a household to understand how different generations experience life in the UK. The first wave of data collection was completed between January 2009 and December 2011. Sampling was conducted using the Postcode Address File in Great Britain and the Land and Property Services Agency list of domestic properties in Northern Ireland, identifying 55,684 eligible households. Interviews were completed with a total of 50,994 individuals aged 16 or older from 30,117 households. At Wave 3, interviews were conducted with 49,768 individuals aged 16 or older from 27,715 households [27]. The current study uses data from Wave 3 only, focusing on 7,685 individuals aged 65 or older. The demographic data are summarised in Table 1. Sample sizes varied across the six regression models due to item-level missingness in the cognitive outcome measures. Each model includes a different cognitive variable as its outcome, and missing observations were excluded listwise. Our key predictor (GHQ-12) and all control variables are consistently available for 7,685 participants. For example, while the full sample included 7,685 participants, the number series task had 6,967 valid observations, indicating approximately 9.3% missingness. GHQ-12 scores were available for 7,681 participants (99.9%). To assess the potential impact of missing data on our findings, we conducted a robustness check by re-running all six models using a balanced sample of 6,967 participants (i.e., the smallest sample size across cognitive outcomes). The results remained statistically similar, suggesting that the missingness did not affect the relationship between cognitive outcomes and GHQ-12. The consistency of results across both full and balanced samples provides reassurance that the missing data are unlikely to be systematically related to our key predictor variable.

**Table 1:** Demographic characteristics of 7,685 participants over 65 years in Wave 3.

Demographic	N	%	SD
Gender			
Female	4,158	54.1	0.50
Male	3,527	45.9	0.50
Ethnicity			
White	7,362	95.8	0.20
Other	323	4.2	0.20
Education			
University degree	1,821	23.7	0.43
Other qualification	3,328	43.3	0.50
No qualification	2,498	32.5	0.47
Marital status			
Married	4,795	62.4	0.48
Divorced	699	9.1	0.29
Widowed	1,798	23.4	0.42
Single	392	5.1	0.05
Children living in the household			
None	7,654	99.6	0.06
One	23	0.3	0.05
Two	8	0.1	0.02
Location			
England	5,687	74.0	0.44
Wales	730	9.5	0.29
Scotland	784	10.2	0.30
Northern Ireland	484	6.3	0.24

Note. Values may not add up to 100% due to rounding or missing values.

### 2.2. Procedures

Data collection was primarily undertaken using Computer Assisted Personal Interviewing, including a self-completion (CAPI).

### 2.3. Measures

#### 2.3.1. Cognitive assessment.

Participants were asked to self-report on their cognitive ability by responding to the following question: “First, how would you rate your memory at the present time? Would you say it is excellent, very good, good, fair or poor?” An additional five cognitive function tests were used in this study and are reported below. Self-reported cognitive ability scores ranged from 1 to 5, while the scores for all other cognitive ability measures ranged from 0 to 100.

#### 2.3.2. Immediate and delayed word recall.

Participants listened to a list of ten words delivered by a computer. They were asked to immediately recall the words, and then again at a later stage without having hearing them a second time. A maximum score of ten correct responses was recorded on each test.

#### 2.3.3. Subtraction.

Participants were asked to subtract 7 from 100 and then subtract 7 again from their answer four more times. A maximum of five correct responses were recorded.

#### 2.3.4. Number series.

Participants were presented with a number sequence in which they populated the gaps in a logical series. Participants were administered two sets of three number sequences. The difficulty of the second set was determined by participants’ performance on the first set. A total score was derived accounting for the difficulty of the items.

#### 2.3.5. Numerical ability.

Participants were given six numerical problems to solve. For example, the first question asked, “In a sale, a shop is selling all items at half price. Before the sale, a sofa costs £300. How much will it cost in the sale?”. Based on participants’ responses, they were administered either one simpler additional problem or two more difficult problems. Correct responses were recorded.

#### 2.3.6. Mental health.

General Health Questionnaire [(GHQ-12; 28)]. The GHQ-12 is a validated screening measure of risk of mental health issues [29]. It comprises 12 items concerning symptoms about general happiness, confidence, the ability to face problems, make decisions, overcome difficulties, and enjoy day-to-day activities over the past four weeks. Six items are worded positively and six are worded negatively. Participants rated their symptoms using a four-point Likert scale relating to the frequency or severity of the symptom compared to what is usual for the respondent (e.g., 1 = more than usual, 2 = about the same as usual, 3 = less than usual, 4 = much less than usual). Scores can range between 0–36 with 36 representing the lowest level of reported subjective well-being. The higher the score, the more likely it is that respondents are suffering from some form of psychological distress. For ease of interpretation, we reversed the overall score so that a value of 36 represents the highest level and going forward we refer to this variable as the mental health variable.

#### 2.3.7. Social Interaction.

Social interaction was measured by the question, ’Do you go out socially or visit friends when you feel like it?’. The answer to this question is binary (Yes, No).

### 2.4. Ethical approval

UKHLS is designed and conducted in accordance with the ESRC Research.

Ethics Framework and the ISER Code of Ethics. The University of Essex Ethics Committee approved Wave 3 of UKHLS. The project received ethical approval at Wave 1 from the National Research Ethics Service (NRES) Oxfordshire REC A (08/H0604/124), at BHPS Wave 18 by the NRES Royal Free Hospital & Medical School (08/H0720/60) and at Wave 4 by NRES Southampton REC A (11/SC/0274).

## 3. Results

### 3.1. Descriptive statistics

A total of 7,685 participants from Wave 3 that met the inclusion criteria were included in the analyses.

### 3.2. Cognition and mental health

Table 2 shows the Ordinary Least Square (OLS) regression coefficients for all cognitive assessment scores. For the regression analyses, we used a cognition variable as an outcome with socio-demographic variables (gender, ethnicity, educational levels, marital status, and number of children) as covariates and GHQ-12 scores as a predictor. We applied this analysis to self-report cognitive ability and for each of the five cognitive tasks.

**Table 2:** Regression analyses with cognitive tasks as outcome, GHQ-12 as predictor and socio-demographic variables as covariates.

Variables	Model 1	Model 2	Model 3	Model 4	Model 5	Model 6
	Self-report Cognitive Ability	Immediate Word Recall	Delayed Word Recall	Numerical Ability	Subtraction	Number Series
GHQ-12	0.05***	0.34***	0.33***	0.31***	0.26**	0.08
	(0.00)	(0.00)	(0.00)	(0.00)	(0.00)	(0.18)
Male	−0.14***	−5.53***	−5.96***	4.54***	5.43***	3.52***
	(0.00)	(0.00)	(0.00)	(0.00)	(0.00)	(0.00)
White	0.13*	7.02***	7.61***	13.39***	6.10**	3.05
	(0.02)	(0.00)	(0.00)	(0.00)	(0.00)	(0.05)
Higher education	0.21***	11.85***	12.12***	15.38***	13.68***	10.44***
	(0.00)	(0.00)	(0.00)	(0.00)	(0.00)	(0.00)
Other education	0.10***	6.67***	7.32***	9.55***	8.27***	2.39***
	(0.00)	(0.00)	(0.00)	(0.00)	(0.00)	(0.00)
Married	−0.05	3.76***	4.20***	4.06***	−0.75	1.78
	(0.36)	(0.00)	(0.00)	(0.00)	(0.66)	(0.20)
Divorced	0.00	2.98**	3.10*	1.60	−1.25	0.05
	(0.94)	(0.00)	(0.01)	(0.19)	(0.54)	(0.98)
Widowed	−0.02	−1.59	−1.97	−0.22	−1.76	−0.35
	(0.75)	(0.09)	(0.07)	(0.84)	(0.34)	(0.81)
1 child	−0.10	3.72	5.52*	3.47	−2.33	3.56
	(0.62)	(0.12)	(0.04)	(0.27)	(0.75)	(0.64)
>1 child	0.31	5.85	13.55**	−12.85***	13.27***	11.99
	(0.57)	(0.20)	(0.01)	(0.00)	(0.00)	(0.58)
Wales	−0.01	−1.04	−0.98	−1.46*	−0.29	0.25
	(0.87)	(0.11)	(0.19)	(0.04)	(0.82)	(0.82)
Scotland	−0.06	−1.89**	−1.25	−1.67*	4.18***	0.69
	(0.10)	(0.00)	(0.09)	(0.02)	(0.00)	(0.51)
Northern Ireland	−0.11*	−1.64*	0.44	1.70	3.80*	3.84*
	(0.02)	(0.04)	(0.64)	(0.08)	(0.02)	(0.01)
Constant	1.58***	32.16***	18.22***	42.60***	58.11***	−4.40
	(0.00)	(0.00)	(0.00)	(0.00)	(0.00)	(0.09)
Observations	7,685	7,619	7,616	7,600	7,385	6,967
F	38.80	78.36	67.70	102.38	34.57	14.04
R^2^	0.06	0.12	0.10	0.17	0.04	0.03

Note: GHQ-12 – general health questionnaire. *** *p* < .001, ** *p* < .01, * *p* < .05.

### 3.3. Self-reported cognitive ability

Results showed that the model was significant with good mental health and the following covariates (female, White, higher educational levels) as significant predictors, F (13, 7671) = 38.80, R^2^ = .063, *p* < .001 (Model 1). Specifically, having good mental health was positively associated with higher self-reported cognitive ability (coef = 0.05, *p* < .001). In terms of the covariate results, being female, being White and having higher education were also significant predictors of self-reported cognitive ability, all *p*s < .02 (see Table 2 for full details). Marital status and number of children at home were not significant predictors (all *p*s > .36).

### 3.4. Individual cognitive tasks

All five individual cognitive tasks (i.e., immediate word recall, delayed word recall, numerical ability, subtraction and number series) were included as outcome variables. Our results showed similar trends for immediate word recall (Model 2), delayed word recall (Model 3), numerical ability (Model 4), subtraction (Model 5), such that a higher GHQ-12 score was positively associated with better cognitive outcomes, all *p*s < .001. Unlike the other cognitive outcomes, having higher GHQ-12 did not significantly predict number series (Model 6) outcome (coef = 0.08, *p* = 0.18), R^2^ = 0.03.

In addition to being female, White, and highly educated, being married or divorced was also a significant predictor of immediate and delayed word recall, and also of numerical ability. Married and divorced individuals performed better on both tasks all *p*s < .001 (Models 2 and 3). For the numerical ability and subtraction tasks (Models 4 and 5), being male was a significant predictor, both *p*s < .001. Other demographics such as being White and having higher education were also significant predictors for Models 2–5. Being White and having higher education was associated with better performance (all *p*s < .001).

### 3.5. Self-reported cognition, mental health, social interaction

Table 3 extends the regression models by including the social interaction variable as a predictor. *Social interaction* was added to determine whether changes in cognitive ability were associated with mental health and social interaction with friends. Socio-demographic variables including gender, ethnicity, educational background, marital status, and number of children were added as covariates. The regression analyses revealed that having higher GHQ-12 scores (coef = 0.04, *p* < .001) and more social interaction with friends (coef = 0.07, *p* = .02) were significant predictors of overall cognitive ability, F (14, 7666) = 36.69, R^2^ = 0.06, *p* < .001 (see Model 7 in Table 3). In terms of covariates, being female, being White, and having higher education levels were all significant predictors (all *p*s < .02). Marital status and number of children in the household were not significant predictors in this model (all *p*s > .31).

**Table 3:** Regression analyses with cognitive tasks as outcome, GHQ-12 as predictor, and social interaction and socio-demographic variables as covariates.

Variables	Model 7	Model 8	Model 9	Model 10	Model 11	Model 12
	Self-report Cognitive Ability	Immediate Word Recall	Delayed Word Recall	Numerical Ability	Subtraction	Number Series
GHQ-12	0.04***	0.27***	0.25***	0.26***	0.24**	0.06
	(0.00)	(0.00)	(0.00)	(0.00)	(0.01)	(0.31)
Social interaction	0.07*	4.99***	5.34***	3.60***	1.27	1.36
	(0.02)	(0.00)	(0.00)	(0.00)	(0.24)	(0.08)
Male	−0.14***	−5.40***	−5.81***	4.63***	5.49***	3.59***
	(0.00)	(0.00)	(0.00)	(0.00)	(0.00)	(0.00)
White	0.13*	6.98***	7.50***	13.35***	6.00**	3.08*
	(0.02)	(0.00)	(0.00)	(0.00)	(0.00)	(0.05)
Higher education	0.21***	11.44***	11.71***	15.11***	13.53***	10.30***
	(0.00)	(0.00)	(0.00)	(0.00)	(0.00)	(0.00)
Other education	0.09***	6.41***	7.04***	9.36***	8.17***	2.32***
	(0.00)	(0.00)	(0.00)	(0.00)	(0.00)	(0.00)
Married	−0.05	3.17***	3.50***	3.63***	−0.97	1.61
	(0.31)	(0.00)	(0.00)	(0.00)	(0.57)	(0.25)
Divorced	0.00	2.57*	2.57*	1.29	−1.44	−0.05
	(0.96)	(0.01)	(0.04)	(0.29)	(0.49)	(0.97)
Widowed	−0.01	−1.73	−2.20*	−0.34	−1.83	−0.38
	(0.78)	(0.06)	(0.04)	(0.76)	(0.32)	(0.80)
1 child	−0.10	3.90	5.69*	3.65	−2.30	3.64
	(0.63)	(0.12)	(0.04)	(0.26)	(0.76)	(0.64)
> 1 child	0.30	5.51	13.17**	−13.09***	13.18***	11.92
	(0.58)	(0.24)	(0.01)	(0.00)	(0.00)	(0.58)
Wales	−0.00	−0.90	−0.85	−1.37	−0.27	0.30
	(0.92)	(0.16)	(0.25)	(0.06)	(0.83)	(0.78)
Scotland	−0.06	−1.95**	−1.33	−1.71*	4.15***	0.70
	(0.10)	(0.00)	(0.07)	(0.01)	(0.00)	(0.50)
Northern Ireland	−0.11*	−1.98*	0.07	1.44	3.70*	3.76*
	(0.02)	(0.01)	(0.94)	(0.13)	(0.02)	(0.01)
Constant	1.54***	30.45***	16.59***	41.39***	57.87***	−4.98
	(0.00)	(0.00)	(0.00)	(0.00)	(0.00)	(0.06)
Observations	7,681	7,615	7,612	7,596	7,381	6,963
F	36.69	79.07	68.97	95.88	32.53	13.34
R^2^	0.06	0.13	0.11	0.18	0.04	0.04

Note: GHQ-12 – general health questionnaire. *** p < .001, ** p < .01, * p < .05.

### 3.6. Individual cognitive tasks, mental health, social interaction

As above, five individual cognitive tasks were assessed as outcome variables.

Results for immediate (Model 8), delayed word recall (Model 9), and numerical ability (Model 10) mirrored those for self-reported cognitive ability such that both having higher GHQ-12 and more interaction with friends were significant predictors of cognitive ability (all *p*s < .001). Similar significant covariates (female, White, higher education) were also significant predictors in Models 8 and 9 (all ps < .001). Compared to singles, being married (both *p*s < .001), or divorced (both *p*s < .004) were also significant predictors in Models 8 and 9. However, for Model 10, being male was a significant predictor (*p* < .001) as well as being White, highly educated and married (all *p*s < .001). For Model 11 with subtraction as a cognitive outcome, the regression analyses showed that having a higher GHQ-12 was a significant predictor (*p* = .01), but not for social interaction (*p* = .24) in this model (). Being male, White and having higher education were also significant predictors in the model 11 (all *p*s < .001). Results for Model 12 (with number series as an outcome) showed that neither GHQ-12 nor social interaction were significant predictors (both *p*s > .08).

## 4. Discussion

We explored associations between cognitive ability and mental health in a large, nationally representative, sample of over 7,000 individuals aged 65 or older. We observed associations between mental health and both self-reported and measured cognitive ability. Better mental health was associated with better cognitive outcomes overall. These results confirm those reported previously [11]. Harada et al. [8] similarly showed that age-related cognitive change is domain-specific, with processing speed and episodic memory declining more than language and vocabulary. This corroborates our findings.

We found positive associations between mental health and all cognitive tasks for those who identified themselves as White compared to non-White and those with higher education compared to those with lower education levels. Studies that have investigated mental health and cognition separately have shown differences due to ethnicity and educational attainment. For example, native-born Whites had significantly better mental health outcomes compared to native-born Blacks [30]. Non-Hispanic Whites had significantly better cognitive scores compared to Hispanics and Non-Hispanic Blacks [31]. Results from a large sample of 502,357 individuals from the UK Biobank cohort demonstrated that higher education was associated with better cognitive performance [32], consistent with our finding that education was a significant predictor across all cognitive domains. Higher educational achievement was also associated with better mental health outcomes in older age [33]. Taken together, it appears that having higher education may serve as a protective factor against cognitive decline.

We also observed gender differences. Positive associations between mental health and cognition were shown for females on the verbal memory tasks (immediate word recall and delayed word recall) and for males on the numerical tasks (numerical ability and subtraction). Previous studies have shown gender differences in cognitive task performance, which appears to extend to the association between mental health and cognitive task performance. For example, a meta-analysis of a large sample of over 40,000 participants reported that females performed better than males in verbal recall tasks [34]. Research supports a female advantage in memory-related tasks, and some verbal subtests. For example, the California Verbal Learning Test and Boston Naming Test reveal differences that persist across the lifespan [24], although we note these differences are generally small. Past research has reported gender differences in mathematical performance but these have narrowed since the 1970s, with males and females performing more similarly on numerical tasks in more recent decades [35]. Kheloui et al. [24] showed a consistent female advantage in verbal memory and male advantage in visuospatial processing across the lifespan. This supports the gender-specific associations observed in the current study and highlights the importance of considering gender-based cognitive patterns when interpreting mental health outcomes in older adults.

Social Role Theory [35] posits that gender differences in cognitive performance may be shaped by societal expectations and unequal access to experiences that promote specific cognitive skills. Empirical evidence supports this view; for example, Hyde [34] emphasises that most gender differences in cognition are modest and largely influenced by social and cultural factors. A recent review reinforces this perspective, highlighting how sex and gender contribute to cognitive variability [22]. The review underscores that observed differences in cognitive abilities are not static or purely biologically determined but interact with contextual, developmental, and psychosocial variables. This theoretical lens helps interpret the gender-specific associations observed in our study and underscores the importance of considering gender as a socially embedded moderator in cognitive ageing research.

Our results showed a stronger association between mental health and performance on the verbal memory tasks for married and divorced people compared to singles. Previous studies that used verbal recall tasks have also reported that singles were at greater risk of memory decline compared to married and divorced people [36]. It appears that being married or living with others, either currently or in the past, has intrinsic qualitative cognitive elements that contribute to improved cognitive engagement and functioning. Regarding mental health, past research has consistently revealed that married people have the lowest prevalence of depression [37], also supported by the current results. Marital status effects can be interpreted through the lens of Social Support Theory [36], which posits that marriage often provides emotional and instrumental support that mitigates the cognitive consequences of psychological distress. Consistent with our findings, married individuals typically benefit from richer social networks and stress-buffering mechanisms, which are protective of cognitive health [37]. However, the protective effects of marriage likely vary by relationship quality and gender, suggesting that marital status alone may not fully capture the complexity of social support in later life.

When we added social interaction to the regression model, most results remained consistent with earlier models (i.e., associations between mental health and self-reported cognitive ability, word recall tasks and numerical ability persisted). However, social interaction was not significantly associated with subtraction or number series performance.. These results suggest that visiting friends had no (or very little) effect on the association between mental health and cognition. There have been longstanding difficulties with measuring the extent of social interaction in older adults. However, it is possible that the particular variable used in this study (a binary response scale) lacked sufficient variability in response options to reveal nuanced individual differences in social isolation in older adults.

The non-significant association between mental health and the number series task may be due to the nature of the number series task, which primarily taps fluid intelligence; the capacity to solve novel problems independent of knowledge [38]. Fluid abilities are more vulnerable to age-related decline [39] and may be less responsive to short-term psychological influences compared to crystallised intelligence, which refers to the ability to use knowledge and experience to solve problems [40]. Tasks that are more reliant on crystallised abilities (e.g., vocabulary, numerical ability) may demonstrate stronger associations with mental health due to these domains being more stable over time and sensitive to the impact of mental health on daily cognitive function [41].

Another possible explanation involves cognitive reserve according to which individuals with higher education or more cognitively enriched experiences are resilient to cognitive decline [42]. Participants with higher education may possess greater cognitive reserve, buffering against the cognitive effects of poor mental health [43], whereas those with lower education may show floor effects in number series performance, masking potential associations with mental health [44]. Cognitive reserve has been linked to better mental health outcomes across the lifespan, including older age. Porricelli et al. [45] found that higher cognitive reserve was associated with lower levels of depression and stress in middle-aged adults. Midlife mental health is a well-established predictor of cognitive outcomes in later life. The protective benefits of cognitive reserve are believed to accumulate over time, with individuals who have higher education entering older adulthood with greater neural efficiency, compensatory cognitive strategies, and psychological resilience [42]. These mechanisms help explain the moderating role of education in the relationship between mental health and cognitive performance observed in our findings.

Studies have found an association between social isolation and poor cognition in people with depression or anxiety [46] suggesting that social networks and engagement in social activities may provide mental stimulation through interactions with others. Our results do not support the idea that social interaction is more beneficial for cognitive abilities in older age. Instead, our results suggest that having good mental health may be more beneficial than social interaction as a protective factor against cognitive decline. Perhaps having good mental health allows individuals to enjoy social interaction more and seek out opportunities for it, compared to those with poorer mental health. However, we often assume that having close social interactions and wider networks brings positive outcomes to the individual, but the social interaction may sometimes lead to negative outcomes such as exposure to stress. This might be particularly prevalent in older adults who may experience ageism and/or discrimination from family members, and this might perpetuate negative stereotypes, thus negatively impacting mental health [47].

Although our findings suggest that good mental health may improve social engagement, it is important to recognise the bidirectional nature of this relationship. Research supports that social isolation can result to mental health issues, but also contribute to them; these, in turn, can negatively impact cognitive functioning. For example, Harada et al. [8] found that loneliness was a predictor of cognitive decline in older adults, and an outcome of mental health decline.

This bidirectional relationship has been increasingly supported by longitudinal evidence. For example, Wang et al. [48] found that social isolation predicted subsequent declines in cognitive function among older adults, with depressive symptoms mediating part of this effect. Lower cognitive function also predicted increased social isolation. Similarly, Huang et al. [49] demonstrated that mental health literacy and social participation were mutually reinforcing in later life, with socio-economic status moderating their effects on active ageing. These findings underscore the importance of considering social interaction not only as a predictor but also as an outcome shaped by mental health status, and they support integrated interventions that target both domains to promote cognitive resilience. Overall, these studies suggest that social isolation exacerbates psychological distress, which in turn impairs cognitive functioning, whereas good mental health fosters greater social engagement.

It is unclear whether mental health decline contributes to cognitive decline, or vice versa. A longitudinal study by Gallagher et al. [12] found that baseline depressive symptoms were significantly associated with greater cognitive decline over time, even after adjusting for demographic and health-related variables. This finding suggests that depressive symptoms may act as a modifiable risk factor for cognitive decline. Despite that, it is possible that early, subtle cognitive changes may lead to depressive symptoms. Future research should prioritise longitudinal designs with multiple waves of assessment.

### 4.1. Strengths and limitations

Our analyses are based on cognitive outcomes collected from a large population-based sample of over 7,000 respondents over 65 years of age providing a high degree of statistical power and good scope for generalisability. Cognitive outcomes were collected at Wave 3 only and therefore represent a single time point only, Although the dataset is longitudinal in design, the current analysis draws on data from a single wave, which limits our ability to examine temporal dynamics or infer causality and limits the use of longitudinal econometric methods such as fixed effects models. Future research should leverage multi-wave data to explore the directionality and evolution of the relationship between mental health and cognitive functioning over time. Additionally, the study is based on correlational evidence only and therefore does not support causal interpretations. Repeated cognitive testing in subsequent waves would allow us to compare our sample across different waves, explore potential changes over time, and rule out cohort effects, which is a limitation of cross-sectional data. Finally, social interaction was measured as a binary variable, which overlooks important nuances such as quality, frequency and subjective experience of loneliness, and therefore reducing sensitivity to subtle social effects. Future studies should incorporate validated scales capturing loneliness, social network size, and interaction quality to better understand their cognitive implications.

### 4.2. Implications

Our findings highlight the relationship between mental health and cognition in older adults, and have important implications for public health and policy aimed at promoting healthy ageing. Better mental health was associated with improved cognitive outcomes, particularly among those who were White, married, and had higher education, pointing to socio-demographic inequalities. Policies should ensure equitable and fair access to mental health support, with a focus on underserved groups. In addition, policies should promote engagement in cognitively stimulating activities to maintain cognitive function. While being married appeared protective, our data did not capture the quality of participants’ relationships or their experiences of loneliness or social isolation (e.g., one may frequently engage socially and feel isolated, and vice versa). Interventions should be tailored to meet the needs of diverse groups. Overall, these findings support a multidimensional approach that integrates mental health support and cognitive health promotion through access to education and socio-culturally informed practices. Such an approach is essential to reducing inequalities and enhance wellbeing in older age.

### 4.3. Conclusion

This study examined the relationships between mental health, cognitive abilities, and socio-demographic factors using a substantial representative population sample of over 7,000 older adults over 65 years old. The study contributes novel evidence by simultaneously examining mental health, cognitive ability, and social interaction within a large, nationally representative sample of older adults. By assessing multiple cognitive domains and integrating socio-demographic factors, our findings offer a more comprehensive model of cognitive ageing than prior studies focused on isolated factors. We found a positive relationship between mental health and cognitive abilities, and that this relationship was impacted by ethnicity, gender, education, and marital status. Mental health plays an important role in maintaining cognitive functioning in older age; however socio-demographic inequalities are prevalent. Gender differences in cognitive task performance suggest that socio-demographic and biological factors impact cognitive ageing. These findings highlight the need for interventions that address both mental and cognitive health in older adults. It is important to provide access to mental healthcare, particularly for underserved groups, alongside promoting education and cognitively stimulating activities to enhance cognitive functioning. A multidimensional strategy is crucial to support mental health, cognition, and social inclusion in older adults.
